# Prediction of protein-protein interactions in dengue virus coat proteins guided by low resolution cryoEM structures

**DOI:** 10.1186/1472-6807-10-17

**Published:** 2010-06-16

**Authors:** Rupali A Gadkari, Narayanaswamy Srinivasan

**Affiliations:** 1 Molecular Biophysics Unit, Indian Institute of Science, Bangalore 560 012, India

## Abstract

**Background:**

Dengue virus along with the other members of the flaviviridae family has reemerged as deadly human pathogens. Understanding the mechanistic details of these infections can be highly rewarding in developing effective antivirals. During maturation of the virus inside the host cell, the coat proteins E and M undergo conformational changes, altering the morphology of the viral coat. However, due to low resolution nature of the available 3-D structures of viral assemblies, the atomic details of these changes are still elusive.

**Results:**

In the present analysis, starting from Cα positions of low resolution cryo electron microscopic structures the residue level details of protein-protein interaction interfaces of dengue virus coat proteins have been predicted. By comparing the preexisting structures of virus in different phases of life cycle, the changes taking place in these predicted protein-protein interaction interfaces were followed as a function of maturation process of the virus. Besides changing the current notion about the presence of only homodimers in the mature viral coat, the present analysis indicated presence of a proline-rich motif at the protein-protein interaction interface of the coat protein. Investigating the conservation status of these seemingly functionally crucial residues across other members of flaviviridae family enabled dissecting common mechanisms used for infections by these viruses.

**Conclusions:**

Thus, using computational approach the present analysis has provided better insights into the preexisting low resolution structures of virus assemblies, the findings of which can be made use of in designing effective antivirals against these deadly human pathogens.

## Background

Dengue viruses (DENV), belonging to the flaviviridae family, are the causative agents of dengue fever and dengue hemorrhagic fever. The four serotypes DENV1, DENV2, DENV3 & DENV4 rely upon *Aedes aegypti *mosquitoes for their transmission between the vertebrate hosts [1]. In the recent past, there had been a resurgence of these viruses as deadly human pathogens with about 50 million infections occurring annually [1]. Yet, no vaccines or specific effective antivirals are currently available. The conventional approach towards vaccine development has not been greatly successful in these viruses[1]. Due to the presence of four different serotypes of the virus, prevention of antibody dependent enhancement (ADE) of the infection has turned out to be rather challenging [2]. Hence, new avenues of vaccine development are being explored [3]. Thus, new knowledge about the potential drug targets can be useful in designing new antivirals.

**Figure 1:**
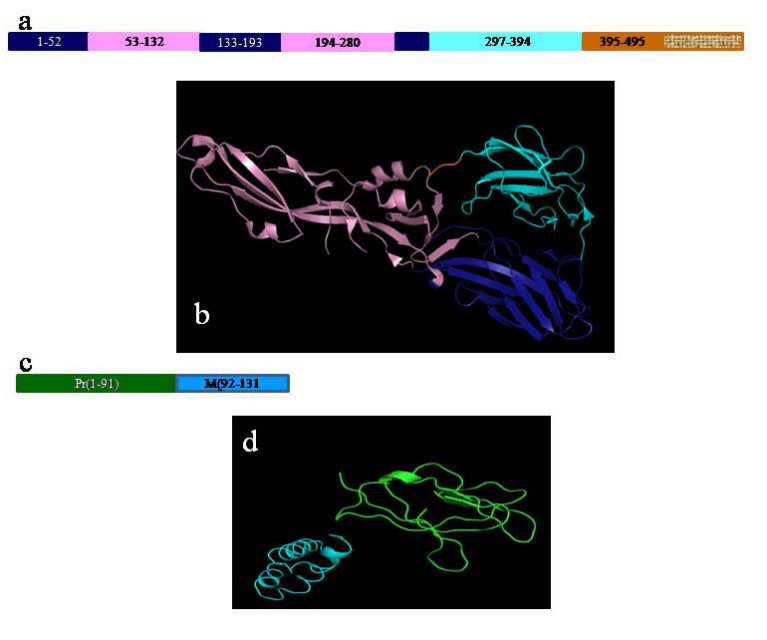
**Domains of E and M proteins**. **1a: **Domain architecture of E glycoprotein - Domain I is shown in blue, Domain II in pink, domain III in cyan and stem region in orange with trans-membrane region shown as textured **1b: **The domains of E are shown in three dimensions using the same color coding as mentioned above (PDB ID 3G7t) **1c: **M protein is expressed along with pre peptide (Pr-shown in green while M in cyan) **1d: **Spatial arrangement of Pr peptide and M protein; shown in Green is the Pr peptide while in cyan is M protein

The coat of the dengue viruses consists of two proteins namely the envelop protein (E glycoprotein) and the membrane protein (M protein) [4]. The E glycoprotein consists of three domains namely a center domain; the domain I, a dimerization domain; the domain II and an immunoglobulin like domain; the domain III [5], as shown in the Figure 1a. In the three dimensional space (Figure 1b), the domain I occupies the central position, hence the name and is flanked by the domain II and the domain III on either side of it. The distal end of domain II comprises fusion peptide [5], which initiates the process of fusion with the host membrane while the domain III has been implicated in binding to the receptors on host cells [4]. The domains I and II are connected by four peptides that serve as flexible hinges while a single peptide connects domain I with III. The coat protein M, as shown in the Figure 1c, is expressed as pre-membrane form (Pr-M) with a glycosylated Pr peptide. During the process of maturation of the viral particle Pr-M undergoes an enzymatic cleavage resulting into the release of Pr peptide (Figure 1d).

Inside the host cell, during the life cycle, the coat proteins E and M of the dengue viruses undergo substantial conformational changes. These changes lead to the changes in their oligomeric states from being heterodimers to homodimers to homotrimers; thus changing the coat morphology while the virus acquires its infectious state [6]. Subsequent to the viral entry in the host cell by receptor mediated endocytosis viral ssRNA is released in the cytosol of host cell. Upon amplification of viral RNA and the synthesis of polyproteins the new viruses start getting assembled in the ER lumen. The newly assembled particles are called as immature viral particles wherein the viral capsid is enclosed in a rough coat. The spiky appearance of the coat is attributed to the arrangement of the heterodimers of E and Pr-M glycoproteins in elevated trimeric fashion. This topology was well captured in cryo-EM fitted model structure of the intact immature viral particle published by Long Li and coworkers [7]. Upon release from the ER lumen and entry into the TGN the coat proteins get exposed to the relatively acidic environment. As a result of this change in the environment, as was observed by Yu and coworkers [8], the trimeric arrangement of E-PrM heterodimers collapses and the heterodimers lie flat on the surface in pairs, in end-to-end fashion. This gives smooth appearance to the coat [8]. The change in the arrangements of these proteins further exposes the furin cleavage site on PrM and facilitates the enzyme action. However, the cleaved Pr peptide remains attached to M protein in acidic pH but is released as soon as the virus is secreted out and exposed back to the neutral environment. The coat of a mature, secreted virus thus comprises M and E proteins and it is believed that these proteins are present as homodimers in the mature, infectious form of the virus. The cryo-EM fitted model structure of mature dengue 2 virus shows primarily homodimers of E-glycoproteins arranged end-to-end [9].

Owing to their importance in the process of maturation of the virus and thus in infection, these coat proteins form attractive targets for designing new drugs. The entire exercise of vaccine development has been immensely benefitted by the knowledge of mechanistic details gained from the plethora of structures that are available of these viral particles at different phases of the life cycle. However, as most of the structures of complete viral assemblies are available only at very low resolutions, the structures provide only Cα traces. Hence, one gets only a course idea about the regions participating in these changing protein-protein interactions. We have developed an objective and automatic method that can recognize/predict protein-protein interaction residues with high sensitivity and accuracy, given the low resolution structures with positions of Cα atoms only [10]. In the present study, using the above mentioned method, we have predicted the residues on both the coat proteins that seemed likely to participate in protein-protein interactions in different phases of the life cycle of dengue virus. The conservation of the inferred interface residues across the flaviviruses was further investigated to attain global picture about the importance of these residues. The present analysis indicated a number of interesting facets of the process of maturation besides giving the residue level details, not reported in earlier analyses of cryo-EM data. The new information reported in this paper will not only impact the present understanding about these viruses but can also be exploited in generating newer and more effective antivirals.

## Results

### Structures of dengue viral coat proteins

In the present study, low resolution structures of dengue viruses have been subjected to the analysis described in our earlier paper [10] to predict the protein-protein interaction interface residues in E and M coat proteins. The interface residues in these low resolution structures were inferred from the Cα coordinates only using the protocol as mentioned in "Methods" section. Briefly, the method for interface recognition is based on the solvent accessible surface area (ASA). Using an unusually large probe sphere the method calculates ASA for every residue in the low resolution protein structure with only positions of Cα atoms available. Using the newly defined residue-specific ASA cutoffs, the method then infers the interface residues with an accuracy of about 84%, sensitivity of above 70% and specificity of above 60%. Based on the distribution of the ASA values of true positives and false positives around the ASA cutoff values in the test dataset, the method also provides confidence measures for every residue that has been inferred to be present in the interface. The confidence score is provided on the scale of one to ten where higher the score higher is the likelihood of the residue being present in the interface. The score of less than three implies low confidence, medium confidence between three and six while the residues with the score above six are inferred to be in interface with high confidence. Using the above mentioned method, three different structures of dengue virus have been analyzed here primarily: the structures of immature dengue 2 virus at neutral pH [PDB: 3C6D][7], acidic pH [PDB: 3C6R] [8] as well as mature dengue 2 virus [PDB: 1P58] [9]. The structures 3C6 D and 3C6R are the models of dengue viral assemblies generated by fitting the high resolution structures of the heterodimeric complexes of coat proteins E and PrM namely 3C5X and 3C6E into cyoEM electron densities[7]. All the three structures consist of three chains of E glycoprotein (A, B & C) and three chains of M protein (D, E & F). Hence, the interactions between all the possible combinations of chain pairs have been investigated using our method as mentioned above. The inferred interface residues of the three structures were then compared to gather better understanding of changes taking place in these interactions, if any, during the process of maturation. The structures 3C6 D and 3C6R contain Pr-peptide (without last 7 residues) of M protein while 1P58 corresponds to the structure of mature M protein without first 20 residues. Hence, for the sake of comparison the numbering in the 1P58 structure for chains D, E and F was modified to show the continuity in the two proteins (Pr peptide and M).

### Protein-protein interactions

As mentioned earlier the dengue virus coat consists of two proteins namely E and M. Hence, the interfaces can be classified as homodimeric or heterodimeric where the homodimeric interfaces are formed by interacting either E or M proteins while the heterodimeric interfaces are formed between a chain of E and a chain of M protein. In the present analysis we have traced these interactions as a function of maturation process of the virus.

### Heterodimeric interfaces

Using the method mentioned above, the residues that are inferred to be participating in the protein-protein interaction interface have been identified. The three structures analysed here consisted of three chains each of E (A, B & C) and M (D, E & F) proteins. Hence, nine different chain pairs were analyzed to obtain the residues contributing to these interactions. The residues from different chains of the E glycoprotein participating in the heterodimeric interactions in all the three structures mentioned above are listed in the Table 1 while the corresponding residues from the coat protein M (Pr-M in immature virus & M in mature) are listed in the Table 2. It is expected that the heterodimeric interactions AD, BE and CE will be identical. Same holds true for the interacting residues in the M protein as well. However, for the sake of completeness, we analyzed all the appropriate combinations of the chains and we pick up a few differences at times, which can be seen in the Tables 1 and 2. These differences are seen in the cases of potentially weak interactions and the putative interface residues are often the border-line cases.

**Table 1:** Heterodimeric interfaces of E glycoprotein

Interacting chains	Immature at neutral pH (3C6D)	Immature at acidic pH (3C6R)	Mature at neutral pH (1P58)
AD	L65 (8.2; h), T68 (7.3; h), T70 (7.3; h), G102 (7.3; h), N103 (7.3; h), H244 (3.5; m), A245 (7.8; h), K246 (6.4; h)	L65 (8.2; h), T68 (7.3; h), T70 (7.3; h), G102 (7.3; h), N103 (7.3; h), A245 (7.8; h), K246 (6.4; h))	F448 (5.6; m), V451 (5.2; m), T454 (5.0; m), M455 (6.9; h), L458 (8.5; h), I459 (5.1; m), I462 (5.1; m)
AE	-	-	-
AF	-	-	-
BD	C74 (8.2; h), C105 (7.8; h), G106 (5.7; m)	-	-
BE	L65 (8.2; h), T68 (7.3; h), T70 (7.3; h), G102 (7.3; h), N103 (7.3; h), H244 (3.5; m), A245 (7.8; h), K246 (6.4; h)	L65 (8.2; h), T68 (7.3; h), T70 (7.3; h), G102 (7.3; h), N103 (7.3; h), A245 (7.8; h), K246 (6.4; h)	F448 (5.6; m), V451 (5.2; m), T454 (5.0; m), M455 (6.9; h), L458 (8.5; h), I459 (5.1; m), I462 (5.1; m)
BF	-	I6 (6.1; h), V151 (7.9; h), G152 (7.4; h), N153 (3.9; m), P364 (7.6; h)	Q494 (3.8; m)
CD	-	-	-
CE	-	I6 (6.1; h), V151 (7.9; h), G152 (7.4; h), N153 (3.9; m), P364 (7.6; h)	Q494 (3.8; m)
CF	L65 (8.2; h), T68 (7.3; h), T70 (7.3; h), G102 (7.3; h), N103 (7.3; h), H244 (3.5; m), A245 (7.8; h), K246 (6.4; h)	L65 (8.2; h), T68 (7.3; h), T70 (7.3; h), G102 (7.3; h), N103 (7.3; h), A245 (7.8; h), K246 (6.4; h)	F448 (5.6; m), V451 (5.2; m), T454 (5.0; m), M455 (6.9; h), L458 (8.5; h), I459 (5.1; m), I462 (5.1; m)

List of residues of E glycoprotein chains (A, B and C) participating in the interactions with M protein chains (D, E and F); confidence scores are given in the brackets where "h" stands for high, "m" for medium while "l" for low confidence

**Table 2:** Heterodimeric interfaces of M protein

Interacting chains	Immature at neutral pH (3C6D)	Immature at acidic pH (3C6R)	Mature at neutral pH (1P58)
DA	L56 (8.2; h), I64 (7.9; h)	T48 (4.7; m), L56 (8.1; h), I64 (5.8; m)	L128 (8.5; h), M136 (8.6; h), I161 (7.9; h), M165 (8.6; h)
DB	-	-	-
DC	-	-	-
EA	-	-	-
EB	L56 (8.2; h), I64 (7.9; h)	T48 (4.7; m), L56 (8.1; h), I64 (5.8; m)	L128 (8.5; h), M136 (8.6; h), I161 (7.9; h), M165 (8.6; h)
EC	-	E60 (5.4; m)	E114 (6.3; h)
FA	-	-	-
FB	-	E60 (5.7; m)	E114 (6.3; h)
FC	T48 (3.7; m), L56 (8.2; h), I64 (6.0; h)	T48 (4.7; m), L56 (8.1; h), I64 (5.8; m)	L128 (8.5; h), M136 (8.6; h), I161 (7.9; h), M165 (8.6; h)

List of residues of M protein chains (D, E and F) participating in the interactions with E glycoprotein chains (A, B and C); confidence scores are given in the brackets where "h" stands for high, "m" for medium while "l" for low confidence

**Figure 2:**
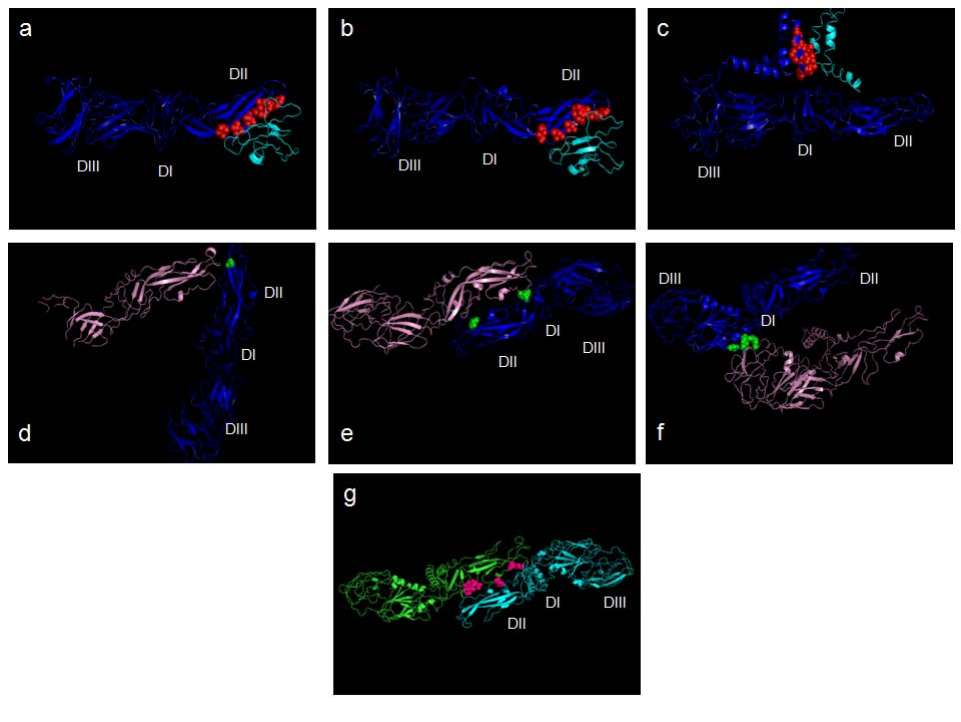
**Pictorial representation of the interface residues of A chain of E glycoprotein 2a, 2b and 2c**. Heterodimeric interfaces of A chain Shown here are the interactions between A chain of E glycoprotein (in blue) and D chain of M protein (in cyan); the interface residues on A chain are shown in red as spheres, Panel a shows the interface in immature virus at neutral pH (structure 3C6D), Panel b shows interface in immature virus at low pH (structure 3C6R) while Panel c shows the interface of mature virus (structure 1P58). Three domains in A chain are labeled as DI, DII and DIII. For comparison sake the three structures were superposed with respect to chain A using Dalilite programme [26,27]. **2 d, 2e and 2f: **Homodimeric interface of A chain Shown here are the interactions between A chain (in blue) and B chain(in pink) of E glycoprotein. The panel arrangement and domain labels are the same as mentioned above in case of 2a, b and c. Interface residues on A chain, participating in homodimeric interface are shown in green color as spheres. The three structures being compared here were superimposed with respect to chain B using Dalilite programme as mentioned above. **2g: **Interface residues of A chain in mature virus (structure 1P58) The residues on A chain (in green) of E glycoprotein interacting with C chain (in cyan) are shown in pink as spheres. The three domains of chain A are labeled as DI, DII and DIII as mentioned above.

Figure 2 is the visual presentation of these interacting residues wherein the interface residues on chain A of E glycoprotein have been highlighted (as a representative) during the course of maturation. For instance, the panels a, b and c of the Figure 2 depict the changes taking place in the inferred interface on A chain of E protein when interacting with D chain of M protein, as the immature virus is exposed to acidic pH (panel b) from neutral (panel a) and subsequently when the virus matures (panel c). As can be seen in the Table 1 and the Figure 2a, b and 2c, in case of E glycoprotein strong heterodimeric interfaces were seen in all the three structures namely that of immature virus at both neutral as well as acidic pH and the mature virus at neutral pH. In the immature virus the heterodimeric interactions appeared to be largely confined to the domain II with a few interactions happening in the domain I, while in the mature form the interactions seemed to have shifted beyond the third domain to either stem region or transmembrane helical region of E glycoprotein. Based on the reports in past, it has been believed that in the mature virus, upon release of the Pr peptide from M protein, the chains of E glycoprotein engage themselves in the homodimeric interactions [7]. However, the results presented here indicated a different picture. In case of M protein, in the immature viruses the Pr peptide seemed to cap the fusion peptide of E protein by interacting with the residues G (102) & N (103) of the fusion peptide of E glycoprotein. However, in the mature virus the M protein seemed to hold the stem region of E and the fusion peptide is left free (as shown in red in the Figure 2c) for interacting with host membrane upon infection. As can be seen in Table 1 and Figure 1, all the seven residues predicted to be participating in heterodimeric interactions in mature virus (F448, V551, T454, M455, L458, I459 and I462) belong either to the stem region or to the transmembrane helix region of E protein. Thus, it appears that even the transmembrane helical regions from the two proteins interact closely, which can be clearly seen in the Figure 2c. Also, these residues were not found to be involved in homodimeric interactions at all.

### Homodimeric interfaces

The homodimeric interactions (either amongst the chains of E or M proteins) were analyzed as a function of viral maturation process. As can be seen in the Table 3 and in the panels d-g of the Figure 2, in case of the E glycoprotein there appeared to be two patches involved in the homodimeric interactions; one covering a part of the domain II close to the fusion loop (Figure 2d and e) and the other more towards the centre of the chain, closer to the domain I in the three dimensional structure (Figure 2f). Interestingly, it was observed that the latter patch possesses PXXP motif and the interactions through this patch seemed to be taking place largely through prolines and glycines (as shown in the Figure 2g). To the best of our knowledge, this is the first indication of the involvement of this motif in protein-protein interactions in dengue viruses. No significant homodimeric interactions were observed in immature virus at neutral pH. In case of coat protein M also there were no significant homodimeric interfaces noted, as reported in the Table 4.

**Table 3:** Homodimeric interfaces of E glycoprotein

Interacting chains	Immature at neutral pH (3C6D)*	Immature at acidic pH (3C6R)#	Mature at neutral pH (1P58)
AB	A245 (7.8; h)	P80 (6.1; h), G223 (3.2; m), D225 (7.0; h)	E133 (1.7; l), P166 (7.5; h), S169 (3.2; m), R188 (6.5; h)
AC	-	-	G78 (3.8; m), P80 (3.8; m), Q86 (2.0; l), L221 (6.1; h), P222 (7.5; h), G228 (3.7; m), N230 (7.2; h)
BA	-	L56 (8.5; h), G78 (3.7; m), V129 (7.9; h), R210 (3.4; m), P222 (7.6; h)	L389 (8.5; h), D390 (7.2; h), W391 (6.9; h)
BC	-	G5 (7.4; h), G100 (7.4; h), F108 (7.3; h), V151 (7.9; h), G152 (7.4; h), L253 (7.5; h), G258 (7.4; h), T315 (7.3; h)	G100 (7.4; h), G102 (7.4; h), F108 (7.3; h), G258 (7.1; h)
CA	-	L389 (6.2; h)	G78 (5.6; m), L221 (6.3; h), P222 (7.5; h), A224 (4.1; m), G228 (7.1; h), N230 (7.3; h)
CB	-	G5 (7.4; h), G100 (7.4; h), F108 (7.3; h), V151 (7.9; h), G152 (7.4; h), L253 (7.5; h), G258 (7.4; h), T315 (7.3; h)	G100 (7.4; h), G102 (7.5; h), F108 (7.4; h), G258 (7.4; h)
	* Interacting chains are arranged in parallel	# Interacting chains are arraged end-to-end	

List of residues of E glycoprotein chains (A, B and C) participating in the interactions amongst themselves. The residues listed in every column belong to the fist chain in the pair of chains listed; confidence scores are given in the brackets where "h" stands for high, "m" for medium while "l" for low confidence.

**Table 4:** Homodimeric interfaces of M protein

Interacting chains	Immature at neutral pH (3C6D)*	Immature at acidic pH (3C6R)#	Mature at neutral pH (1P58)
DE	-	-	-
DF	-	-	-
ED	-	-	-
EF	M37 (9.0; h), G42 (7.4; h)	-	L153 (8.6; h), A160 (7.8; h)
FD	-	-	
FE	M37 (8.6; h), G42 (5.7; m)	-	L153 (8.6; h), A160 (7.8; h)
	*Interacting chains are arranged in parallel	#Interacting chains are arranged end-to-end	

List of residues of M protein chains (D, E and F) participating in the interactions amongst themselves. The residues listed in every column belong to the first chain in the pair of chains listed confidence scores are given in the brackets where "h" stands for high, "m" for medium while "l" for low confidence

### Conservation of protein-protein interaction interfaces

**Figure 3:**
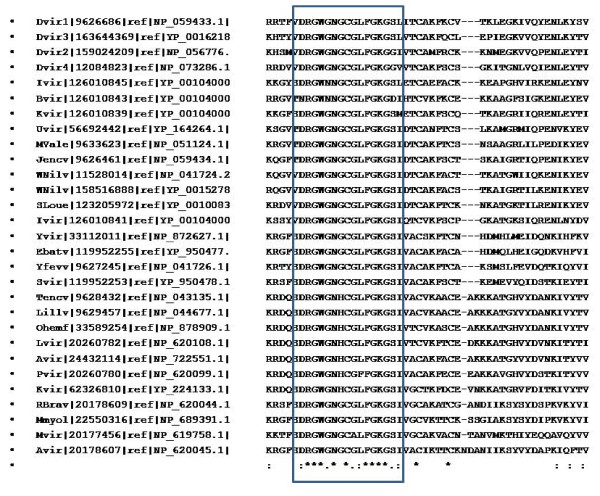
**Conservation of the fusion peptide in E glycoprotein**. Shown here is the multiple sequence alignment of E glycoprotein across different members of Flaviviridae family, generated using ClustalW program. Marked with the rectangle is the fusion peptide region in this alignment. (*) at the bottom of the column indicate complete conservation of the residues.

**Figure 4:**
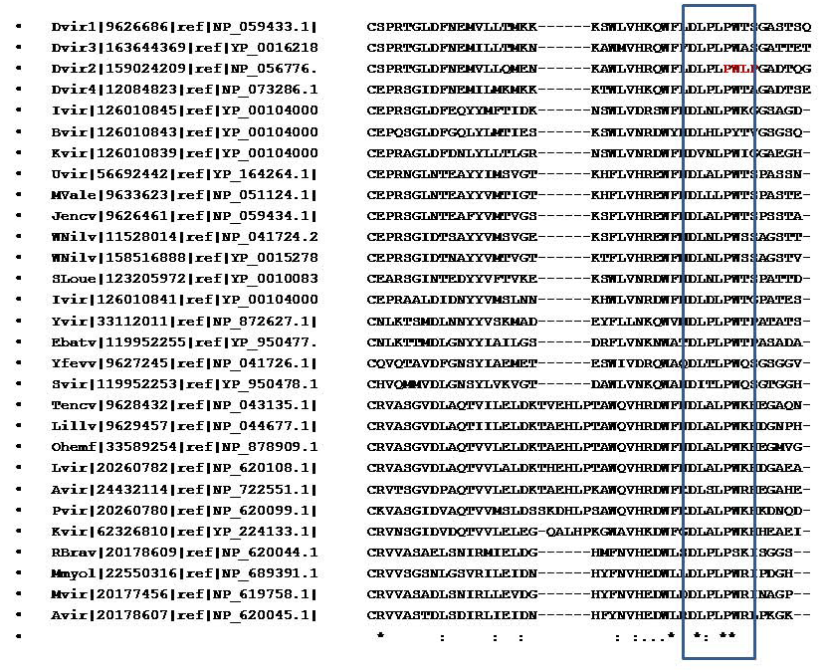
**Conservation of proline-rich motif in E glycoprotein**. Shown here is the multiple sequence alignment of E glycoprotein across different members of Flaviviridae family generated using the same method as mentioned above and the proline-rich motif is marked with a rectangle. (*) at the bottom of the column indicates complete conservation of the residue while (:) indicates conserved substitution at that position. The "PWLP" motif in Dengue 2 is shown in bold.

**Figure 5:**
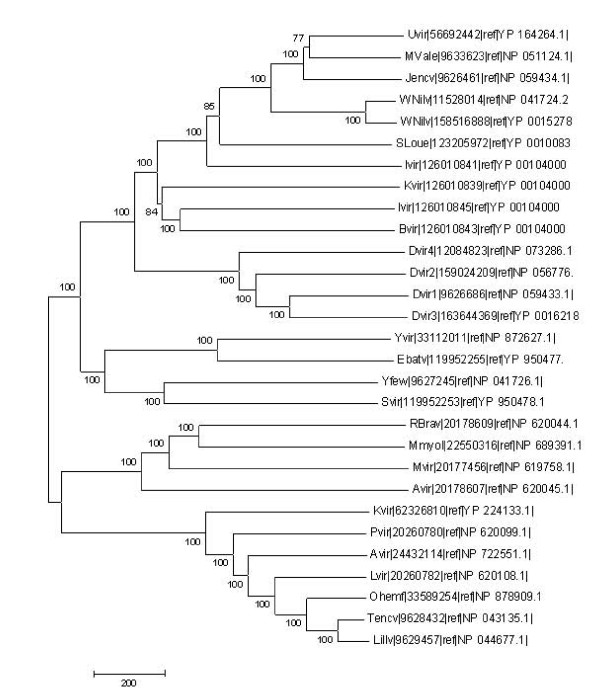
**Phylogenetic analysis of E glycoprotein**. The figure displays phylogenetic tree diagram for E glycoprotein. The multiple sequence alignment of E glycoprotein across the members of flaviviridae family was used to generate the phylogenetic tree using neighbor joining method.

The viruses belonging to the flaviviridae family are known to utilize similar mechanisms for infecting the hosts although they use different arthropod vectors for their transmission and these common features have been targeted for vaccine development against this family of viruses [11]. Hence, the conservation of the residues that were inferred to be participating in the protein-protein interactions in dengue coat proteins was investigated. The multiple sequence alignments generated for the E glycoprotein revealed that the fusion loop is maximally conserved amongst the members of the flaviviridae family (Figure 3). The residues of domain II participating in protein-protein interactions were found to be better conserved than those belonging to the domains I and III. The PXXP motif was not seen to be conserved in most of the members of flaviviridae except for only two namely Yokos virus and Entebbe bat viruses for which the arthropod host is not known (Figure 4), indicating the possibility of the existence of species specific mechanisms of infection within the flaviviridae family. Detailed phylogenetic analysis (Figure 5) revealed that the above mentioned two species (Yvir and Ebatv in the figure 5) are evolutionarily close to dengue viruses (Dvir1 to Dvir4), even closer than other members such as yellow fever virus (Yfew), West Nile (Wnilv) virus that use mosquitoes as vectors just like dengue viruses.

## Discussion

The coat proteins E and M of dengue viruses are being targeted for the development of new antivirals. During the maturation process of the viral particles, the two proteins interact closely and undergo conformational changes leading to the change in the morphology of the viral coat. However, details of the interactions between the two proteins have been elusive as most of the structures of the viral assemblies are available at very low resolutions. In the present analysis, by using a newly developed method which combines recognition and prediction, the interface residues of the two proteins in the viral assemblies have been predicted from these low resolution structures with only Cα atom positions available. Good conservation of these residues supports our identification. A comparative analysis of the three different structures of dengue viruses at different phases of their life cycle pointed some of the interesting details of the process of maturation.

In the immature viruses, where the coat proteins E and M form heterodimers that are arranged as trimers, the M protein appeared capping the fusion peptide of the E glycoprotein. The two residues of the fusion peptide namely G(102) and N(103) seemed likely to interact with Pr peptide of the Pr-M directly, thereby preventing premature fusion with the host membrane. These likely interactions were found to be present in immature viruses, both before and after the furin cleavage. The two residues were found to be conserved across the flaviviridae members thus reiterating the fact that all the members must be using common mechanism for the fusion with the host membrane for the release of the nuclear material. Although, this mechanism had been reported earlier in the literature [5], the present study points out the residues likely to be interacting in the process of membrane fusion.

As the immature viral particles get transformed into the mature, infectious particles, it is believed that the E and M proteins transform from being heterodimers (E-PrM) to homodimers (E-E & M-M). However, the present analysis revealed a possibility of heterodimeric interfaces being present even in the mature viruses (Figure 2c). In the immature phase the heterodimeric interfaces seemed likely to be present mainly on domain II of E and Pr peptide of M. However, in the mature form E and M proteins seemed interacting only near the stem regions (distal to domain III in E) and transmembrane helices, where no homodimeric interfaces were noted. Thus, the protein-protein interactions in the coat of mature dengue virus can be imagined to be present in two tiers, the lower tier formed by stem and transmembrane regions that are engaged in heterodimeric interfaces while the upper tier formed by the three domains in case of E, that are engaged in homodimeric interfaces. Interestingly, no significant homodimeric interfaces were located in the immature viruses before furin cleavage (at neutral pH). A few intermediate homodimeric interfaces were spotted in the immature virus post furin cleavage (at acidic pH). The presence of these likely interactions were also confirmed in the other structures of dengue viruses namely the structure of mature dengue virus surface in complex with the carbohydrate recognition domain of DC-SIGN [12] [PDB: 2B6B] and the structure of immature virus [PDB: 1TGE) [13]. However, in case of the structure of the mature virus bound to the neutralizing antibody [PDB: 2R6P] the above mentioned interactions were found to be disrupted, perhaps owing to the conformational changes brought about by the bound antibody [14].

In case of mature dengue 2 virus the two chains of E glycoprotein seemed interacting partly mediated by PXXP motif. The PXXP motif and other proline-rich sequences are known to be involved in protein-protein interactions [15,16]. In case of dengue virus 2 the region close to PXXP motif has a few more prolines nearby. Although, the PXXP motif is not completely conserved in most of the flaviviruses, the other prolines in the region appeared largely conserved. Hence, there exists a possibility of either structure driven interactions in this region or species specific mechanisms of infection within the family of flaviviridae. In case of HIV-1 the involvement of PXXP motif in the interactions with SH3 domains of some of the Src kinases has earlier been reported [17]. Interestingly, in the case of dengue 2 virus the homodimeric interactions through this motif were detected only in the mature viruses. Thus, involvement of this motif in the interactions with the other host proteins in the immature viruses is a distinct possibility which remains to be explored further. In case of *Mycobacterium tuberculosis *and *Plasmodium falciparum *the therapeutic implications of PXXP motifs are being tested [18]. Such a possibility in case of dengue viruses needs to investigated.

## Conclusions

In the present analysis, use of the computational methods has enabled us to predict some of the finer details from the available low resolution structures of dengue virus assemblies. Our newly developed method, based on the accessibility criterion, has predicted the protein-protein interaction interface residues on dengue virus coat proteins with significantly high confidence, solely from the Cα atom positions in cryoEM structures. Besides "predicting" the interfacial residues known already from earlier studies the present analysis provides an extended list of interfacial residues. Further, the present work recognizes significant changes in the assembled structures of coat proteins, as a function of maturation of the virus. For the first time, in dengue virus, involvement of proline-rich regions in protein-protein interactions has been suggested. The main findings of the present analysis can have significant influence in human intervention of disease processes mediated by dengue and closely related viruses.

## Methods

### Structures analyzed

The PDB codes [19] and descriptions of the cryoEM fitted models analyzed in the present study are as follows:

1. 3C6 D: Immature DENV 2 at neutral pH [8]

2. 3C6R: Immature DENV 2 at acidic pH [7]

3. 1P58: Mature DENV 2 [9]

4. 2B6B: Mature DENV 2 bound to carbohydrate recognition domain of DC-SIGN [12]

5. 2B6R: Mature DENV 2 bound to a neutralizing antibody [14]

6. 1TGE: Immature DENV 2 [13]

### Recognition of protein-protein interaction interfaces

The new method developed in house was used to infer protein-protein interaction interfaces in the low resolution structures giving Cα atom positions only [10]. Briefly, the method mimics the popular approach used for protein-protein complex structures with all the atomic positions available and using the solvent accessibility calculations [20]. The accessible surface area values in these structures have been calculated using probe of larger probe radius (of 3.5Å radius instead of default 1.4Å). In the high resolution structures a residue is said to be present in the interaction interface if it is completely buried in the complex form (Accessibility < 7%) and exposed in the isolated form (Accessibility > 10%) [21]. In our method we have defined the residue level cutoff values in terms of accessible surface area values that are corresponding to 7% and 10% accessibility values. Using these limits the residues were then inferred to be participating in the protein-protein interactions. A confidence score has been provided for every residue that was designated as interface residue. The confidence score for a residue was calculated based upon the distribution of the ASA values of the true positives and the false positives in our test dataset around the ASA cutoff value for that residue. The scores range on the scale of one to ten where score of less than three implies low confidence (l) in the residue being a true positive while score above six indicates high confidence in designating the residue as interface residue. The scores ranging from three to six imply medium confidence in inferring the residue as interface residue (m). In the structures determined at very low resolutions the assignments of even Cα atom positions are likely to be error prone. Hence robustness of the method was determined for a few case study examples. The method used to assess the robustness and the results obtained in this case are summarized in the Additional file 1. As can be seen clearly from the Additional file 1 table S1 the average robustness factor is about 63% and hence, our method is reasonably robust to the inaccuracies in Cα trace.

### Numbering scheme for M protein

The sequences of M protein from different structures of dengue viruses were aligned with the sequence of polyprotein as given in NCBI and a numbering in structure 1P58 was modified to bring about the continuity in counting as used in case of 3C6 D and 3C6R

### Multiple sequence alignments

The homologues of E and M proteins in other viral species were identified by carrying out sensitive search using PSI-BLAST [22] against all the viral genomes available till date. The sequences showing greater that 30% sequence identity with the query sequence were selected for further usage. Subsequently multiple sequence alignments were carried out amongst the selected sequences using ClustalW [23].

### Phylogenetic analysis

Using the multiple sequence alignments generated as mentioned above, a detailed phylogenetic analysis was carried out using MEGA software version 4 [24,25]. The trees were obtained using Neighbor joining method and the reliability of the trees was tested by using Bootstrap test of phylogeny (1000 replicates).

## Authors' contributions

1] RG designed the problem and contributed substantially in acquisition and analyzing the data and interpretation of results besides drafting the article and revising it and approving the version to be published. 2] NS contributed substantially in the analysis of data as well as interpretation of results besides contributing to drafting the article and revising it critically for important intellectual content as well as final approval of the version to be published.

## Supplementary Material

Additional file 1 **To investigate robustness of the method used for prediction of protein-protein interaction interface residues**. The additional file provides details of the method used to calculate robustness of the method and a table (Additional file table S1) summarizing the results obtained. Click here for file
