# Supplementary material for: Prediction of protein-protein interactions in dengue virus coat proteins guided by low resolution cryoEM structures
Source: BMC Struct Biol. 2010 Jun 16;10:17. doi: 10.1186/1472-6807-10-17 (PMC2906493; doi:10.1186/1472-6807-10-17)
Supplement: Additional file 1 — To investigate robustness of the method used for prediction of protein-protein interaction interface residues. The additional file provides details of the method used to calculate robustness of the method and a table (Additional file table S1) summarizing the results obtained. [file 1472-6807-10-17-S1.DOC]

Additional information

Here we report our assessment of the robustness of the method used for prediction of protein-protein interaction interface residues based on the trace of Cα atoms in the low resolution cryoEM structures. We have addressed the robustness of identification of exposed, buried and interfacial residues considering the low accuracy of positions of Cα atoms in the low resolution structures.

Method:

In order to assess the sensitivity of our method to the inaccuracies in the Cα trace, we have calculated robustness for our method taking five different case study examples with total of 16 different pairs of subunits. Towards this following method was followed:

a] In every pair at least two different structures of a multi-subunit protein, one at high resolution and other at very low resolution were identified.

b] The interface/ buried residues for a particular chain/subunit were then predicted in both the structures using our method that uses Cα atom positions only (as mentioned in the main manuscript as reference number 10).

c] The lists of interface/buried residues in the above mentioned two cases and were compared and robustness factor was calculated using following formula:

2 X number of common residues between the two lists

Robustness factor (%) = ^____________________________________________________________^ X 100

Total number of residues in two lists

Scan of the structures in the protein data bank did not yield us significant number of examples where we could compare the interfaces. However, as our predictions of interface residues critically depend on our ability to predict buried/surface exposed residues, expressing robustness in terms of sensitivity with recognition of buried residues will in a way reflect robustness of the method. Hence, in our result table we have more examples of robustness expressed in terms of buried residues than interface residues.

The results obtained are listed in the Additional file table S1 below.

Additional file table S1: Robustness of the method used for interface determination

Table summarizes the robustness factor values for 5 case study examples. As mentioned in method, robustness factor is expressed either in terms of interface residues or buried residues

|  | Structure 1 (resolution Å) | Structure 2 (resolution Å) | Robustness factor (%) |
| --- | --- | --- | --- |
| Case 1 (interface) | 3CAU_A (4.2)  3CAU_B (4.2)  3CAU_C (4.2)  3CAU_D (4.2) | 2NWC_A (3.02)  2NWC_B (3.02)  2NWC_C (3.02)  2NWC_D (3.02) | 40.0  61.1  61.1  76.1 |
| Case 1 (buried) | 3CAU_A (4.2)  3CAU_B (4.2)  3CAU_C (4.2)  3CAU_D (4.2) | 2NWC_A (3.02)  2NWC_B (3.02)  2NWC_C (3.02)  2NWC_D (3.02) | 50.5  50.8  49.9  51.2 |
| Case 2 (buried) | 1XI4_A (7.9)  1XI4_B (7.9)  1XI4_C (7.9) | 1BPO_A (2.6)  1BPO_B (2.6)  1BPO_C (2.6) | 68.5  68.4  68.9 |
| Case 3 (buried) | 1XI4_A (7.9)  1XI4_B (7.9) | 1C9I_A (2.9)  1C9I_B (2.9) | 76.8  75.5 |
| Case 4 (buried) | 1XI4_A (7.9)  1XI4_B (7.9) | 1UTC_A (2.3)  1UTC_B (2.3) | 74.8  73.5 |
| Case 5 (buried) | 1XI4_A (7.9) | 1B89_A (2.6) | 61.8 |

The overall robustness factor is about 63%.
